# Spoonerism Beyond Language: A Multi-Componential Perspective on Phonological Awareness

**DOI:** 10.3390/brainsci15080878

**Published:** 2025-08-18

**Authors:** Francesco Benso, Noemi Mazzoni, Carlo Chiorri, Eleonora Ardu, Paola Venuti, Angela Pasqualotto

**Affiliations:** 1 Department of Psychology and Cognitive Science, University of Trento, 38122 Trento, Italy; 2 Department of Theoretical and Applied Sciences, eCampus University, 22060 Novedrate, Italy; 3 Department of Education Sciences, University of Genoa, 16126 Genoa, Italy; carlo.chiorri@unige.it; 4 Associazione Neuroscienze Cognitive Clinica Ricerca Intervento (ANCCRI), 10138 Torino, Italy; 5 Faculty of Psychology and Education Sciences (FPSE), University of Geneva, 1205 Geneva, Switzerland; 6 Department of Education and Learning, University of Applied Sciences and Arts of Southern Switzerland, 6600 Locarno, Switzerland

**Keywords:** spoonerism, reading abilities, phonological awareness, dynamic modularity, multi-componential task

## Abstract

**Background:** Reading difficulties are closely linked to phonological awareness (PA), though PA tasks vary in complexity and cognitive demands. Recent research suggests that dyslexia reflects multiple cognitive risk factors, aligned with multi-level models of reading and recent theories of complex modularity. These models propose that different tasks engage different cognitive modules depending on their structure, according to a dynamic and graded organization. **Methods:** This study investigates cognitive functions that predict performance on a complex PA task (spoonerism) in 115 fourth-grade Italian students. **Results:** The results indicate that: (1) dividing the sample into high- and low-performing groups in verbal working memory (alpha span test) and visuospatial working memory (object updating task) revealed that students with lower working memory capacity performed significantly worse on the spoonerism task—underscoring the importance of general working memory for this type of activity; (2) Gaussian graphical models showed that spoonerism performance was strongly associated with the object updating task (r = 0.47) and the alpha span test (r = 0.33), confirming a close link between this phonological task and general working memory. **Conclusions:** These findings support the view that complex PA tasks depend on a broader set of cognitive systems beyond phonological processing. They align with theories of dynamic modularity, which propose that modularity arises from task demands, not fixed anatomical constraints. In children, the involvement of executive attention suggests that such tasks are not yet automatized but rely on central cognitive control. Understanding this complexity is crucial for interpreting reading performance and developing targeted, multi-componential interventions.

## 1. Introduction

Reading acquisition is one of the most complex achievements of childhood, requiring the coordination of multiple cognitive and linguistic systems. Among the most robust predictors of reading development is phonological awareness (PA)—the capacity to detect, access, and manipulate the sound structure of spoken language [1,2]. This skill is crucial for establishing the mapping between graphemes and phonemes, and its role in reading acquisition has been extensively documented across orthographies [3].

Complex PA tasks like spoonerisms —where individuals are asked to swap the initial sounds of two spoken words (e.g., turning “cat boat” into “bat coat”)—are frequently used in both educational and clinical settings and are often interpreted as pure measures of phonological skill. However, emerging theoretical and empirical work suggests that interpreting spoonerism purely as a phonological awareness task may be overly simplistic. PA is not a unitary skill. Tasks used to assess PA vary substantially in complexity, ranging from phoneme identification and syllable segmentation to more demanding manipulations such as phoneme deletion or spoonerism [4]. These tasks differ not only in surface difficulty but also in the types of cognitive processes—both phonological and domain-general—that they engage. For instance, spoonerism tasks require not just access to phonological representations but also working memory, attentional control, and response inhibition [5,6,7]. This aligns with Baddeley’s multi-component model of working memory, which emphasizes the central executive’s role in coordinating the manipulation of verbal information [8,9,10]. As such, they can be seen as composite cognitive tasks, drawing on both domain-specific and domain-general systems. This complexity becomes particularly relevant for understanding developmental dyslexia, which has traditionally been explained in terms of phonological deficits but may also involve broader cognitive challenges.

A substantial body of evidence has linked phonological awareness with developmental dyslexia. The phonological deficit hypothesis posits that dyslexia stems from impairments in the representation, access, and manipulation of phonological information, which in turn hinder the acquisition of grapheme–phoneme correspondences [11,12,13]. This hypothesis is supported by extensive cross-linguistic evidence showing that children with dyslexia perform poorly on a wide range of PA tasks [14] and that early PA skills are strong longitudinal predictors of reading difficulties [15,16]. However, growing evidence indicates that PA impairments are neither necessary nor sufficient for dyslexia in all cases [17,18,19]. For instance, some children with dyslexia exhibit intact phonological awareness but impaired rapid automatized naming or visual attention span [20,21,22], while others with weak PA still acquire accurate decoding skills through compensatory mechanisms [23,24], e.g., reliance on semantic or orthographic cues. These findings suggest that dyslexia reflects multiple cognitive risk factors rather than a single core deficit, prompting the development of multi-factorial models [25,26]. Within these models, complex PA tasks such as spoonerism can be particularly informative, as they are sensitive to both phonological and domain-general weaknesses and can help distinguish subtypes of dyslexia characterized by broader cognitive vulnerabilities.

In line with this, several scholars have argued for a multi-componential view of PA. For example, ref. [6] distinguishes between simple PA tasks, such as syllable blending, and complex tasks, such as phoneme deletion and spoonerisms, which involve heavier working memory and executive attention demands. The complexity of these tasks has raised questions about whether performance reflects purely phonological skill or rather the interaction between phonological and general cognitive abilities [27]. This aligns with findings from other studies suggesting that working memory significantly predicts performance only on high-load PA tasks, while simple PA tasks may be less dependent on executive resources [28].

These findings support the notion that spoonerism tasks, far from being purely linguistic, tap into a broader cognitive architecture—highlighting the need to reconceptualize them as multi-componential tools for cognitive profiling. Understanding the nature of these interactions calls for a deeper theoretical framework. Cognitive modularity offers one such framework, originally conceptualized by Fodor [29] as the idea that the mind consists of specialized, informationally encapsulated systems. In this framework, encapsulation refers to the idea that modules operate independently of information from other systems, while mandatoriness denotes their automatic and involuntary activation. However, rigid modular accounts have since been challenged by more dynamic models of complex systems. Anderson’s neural reuse theory [30], for instance, posits that brain regions are not uniquely dedicated to specific cognitive functions but are reused in multiple tasks. Barrett and Kurzban [31] similarly argued that modularity is not anatomical but functional: multiple overlapping systems are recruited flexibly in response to different contexts and computational challenges. Other paradigms, though conceptually distinct, support more open and nuanced views of modularity. Examples include the theory of massive modularity [32] and the dynamic networks described by mirror neuron researchers [33].

This conceptual shift toward dynamic modularity is particularly relevant for reading. Neurocognitive studies show that a single brain region may support multiple functions depending on task demands. For example, the visual word form area (VWFA), located in the left lateral occipitotemporal sulcus, is consistently activated during reading tasks but is also functionally connected to both linguistic and visuospatial networks [34,35]. This supports the view, proposed Mahon and Cantlon [36], along with Rabaglia et al. [37], that cognitive systems can be classified along a continuum—from fully encapsulated modules to more flexible, partially automatic systems like reading (see Table 1).

A foundational taxonomy for such classifications was developed by Moscovitch and Umiltà [38] and recently revised by Benso et al. [39], arguing that modularity in reading should be understood as a functional property shaped by learning and context. We begin by summarizing this taxonomy (Table 1) and then apply it to the reading module to support our central hypothesis: that the reading system develops as a multicomponential architecture involving both domain-specific and domain-general components.

**Table 1:** Key properties of modularity of cognitive systems, adapted from [39] and inspired by [38].

Modularity Level	Examples	Impairments	Key Features
First-level modules	Visual and auditory frequency perception, motor reflexes	Agnosia, auditory agnosia	Genetically innate; fully encapsulated; domain-specific; mandatory operation [29].
Second-level modules	Object recognition, first-level language, walking	Aphasia, basic agnosias	Genetically predisposed but require environmental input; partially encapsulated; domain-specific; semi-mandatory operation.
Third-level modules	Reading, complex motor skills (e.g., dancing)	Dyslexia, alexia, apraxia, agraphia	Assembled through experience and hyper-learning; during development, they depend on executive and attentional control (CEN); exhibit low encapsulation; interact across domains; show functional specialization, semi-mandatory operation.
**Additional Characteristics of the Model** State Flexibility Second- and third-level modules can alternate between a modular, mandatory state and a non-modular state governed by executive attention, depending on task demands and cognitive context (e.g., [40,41,42]). Core Principle of the Model Mandatoriness is intrinsically linked to encapsulation and follows a gradient of automaticity: it progressively decreases from first- to third-level modules. In third-level systems, automaticity is highly dependent on expertise and task familiarity. A minimal degree of mandatoriness—even if partial or context-dependent—is a necessary condition for classifying a complex system as modular.			

In parallel, we examine what PA tasks like spoonerism actually measure. While these tasks involve multiple components, we argue they do not meet criteria for modularity. Rather, their successful execution relies heavily on domain-general systems—particularly working memory and executive attention.

As argued by [38] and illustrated in Table 1 [39], reading is classified as a third-order module, characterized by specific functional properties that may partially account for the strong and well-documented association between PA, spoonerism tasks, and reading ability.

Some investigations have also examined bilingual contexts to show how phonological awareness constitutes a fundamental and cross-linguistic component. The cross-linguistic stability of phonological awareness as a key component in reading development has been documented in both monolingual and bilingual children, including those with diverse language backgrounds and orthographic systems. For example, phonological awareness has been shown to predict reading accuracy in Sylheti–English bilinguals [43] as well as in Italian–Arabic and Italian–Romanian bilinguals [44]. These findings suggest the presence of a shared underlying mechanism for reading acquisition across languages, despite structural differences. Crucially, this foundation appears to involve not only language-specific phonological skills but also the coordination of attentional and working memory resources.

This perspective aligns with evidence from studies on children with dyslexia, which show that the link between working memory and reading is mediated by phonological awareness, especially in tasks with high cognitive demands such as spoonerisms [7].

Tasks like spoonerisms, although traditionally considered phonological, may recruit distinct modular or submodular components such as verbal and visual working memory, executive attention, or attentional control depending on the level of challenge and familiarity. This perspective aligns with multi-level models of reading and PA (e.g., [45,46]), which propose a hierarchy of modules (see Table 1) and submodules that are differentially activated depending on task demands.

**Figure 1:**
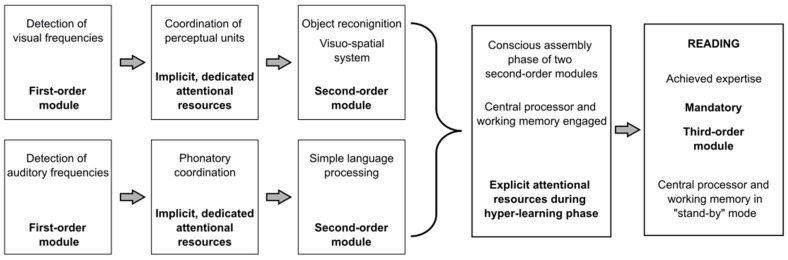
Schematic representation of hierarchical modularity in reading acquisition. The figure shows how first-order modules feed into second-order modules, which are consciously assembled via executive resources during a hyper-learning phase, culminating in an automatized third-order system.

Building on the modularity framework proposed by [39] and grounded in the work of [38], we introduce a schematic model (Figure 1) to illustrate the developmental progression of the cognitive and neural systems that support reading acquisition.

As illustrated in Figure 1, reading is a multi-componential system that integrates multiple domain-specific and domain-general processes through executive attention and mechanisms of hyper-learning. Following [47], it should not be conceptualized as a strictly domain-specific ability but rather as a functionally specialized system emerging from the interplay of distinct cognitive domains (see also Table 1).

Once automatized, third-order modules—like reading—can operate with reduced reliance on executive resources. However, as noted by [39], executive attention remains in a “standby” state, ready to intervene when task demands or environmental conditions change. Each transition toward a higher-level module in Figure 1 implies a decreasing degree of automatization and an increasing reliance on coordination mechanisms. At the same time, third-order modules must reach a sufficient degree of mandatoriness—determined by the level of acquired expertise—in order to be considered modular.

Spoonerism tasks appear to involve cognitive processes that resemble those required during early reading acquisition. In these tasks, children must hold a mental representation of the words in working memory, manipulate their initial phonemes—i.e., mentally switch them to produce novel phonological sequences. This process relies on the transformation of visual–orthographic input into phonological output via domain-general mechanisms, particularly working memory and attentional control. Such demands may explain the strong correlation consistently observed between spoonerism performance and reading ability.

However, unlike reading, spoonerism tasks function as experimental tools rather than learned skills and therefore do not meet the criterion of mandatoriness that defines modular systems. As such, while they draw on some of the same cognitive systems as reading, they should not be considered modular processes in themselves.

These considerations suggest that spoonerism performance reflects executive attention and working memory more than pure phonological ability and that these domain-general components are likely central to what the task actually measures. To investigate this issue empirically, we designed a study to clarify the cognitive underpinnings of spoonerism tasks.

Our theoretical synthesis integrates insights from developmental, cognitive, and clinical research—focusing on literature that addresses the cognitive underpinnings of complex PA tasks across both typical and atypical populations. From a developmental perspective, dynamic modularity is especially relevant: children’s success in these tasks may reflect not only phonological skills but also their broader cognitive profiles. For example, the central executive system—responsible for manipulating and updating information—has been shown to be involved in spoonerism performance [7,46]. This makes spoonerism an ideal lens through which to examine the interplay between linguistic and executive systems in childhood.

However, to our knowledge, few studies have systematically investigated how variability in domain-general abilities explains performance in phonological awareness tasks among typically developing children. This study addresses this gap by providing an empirical investigation of the cognitive components involved in spoonerism tasks, with a specific focus on more domain-general processes. Rather than limiting our sample to children with dyslexia, we assessed a large group of Italian fourth graders to explore how individual differences in cognitive abilities contribute to performance variability.

We hypothesized that performance on the spoonerism task would not be explained solely by phonological processing (*H*_0_) but would instead reflect the contribution of domain-general cognitive systems such as working memory and executive attention (*H*_1_). If this is the case, it would support the interpretation of phonological awareness as a multi-dimensional, composite skill rather than a purely linguistic one.

## 2. Materials and Methods

### 2.1. Participants

A total of 115 fourth-grade Italian children between 8 and 9 years of age participated in the present study. We decided to focus only on fourth graders because at this age the reading abilities are well-established and children are able to perform complex tasks with high executive load [48] such as the spoonerism task. The children were recruited through two primary schools in the urban area of Genoa (northwest Italy). All children were Italian native speakers with at least two years of literacy instruction and normal or corrected-to-normal vision and hearing abilities. Children with psychological, neurological, or medical diagnoses, such as ADHD, autism spectrum disorder (ASD), epilepsy, or other relevant neurological and medical conditions were not included in the study. All participants and their parents received a detailed explanation of the procedure and provided their signed informed consent, in accordance with the Declaration of Helsinki.

### 2.2. Procedure

All children underwent a battery of tasks designed to vary in WMC demands, progressing from those with high cognitive load to those with minimal load—that is, from less modular to more modular tasks. This sequencing allowed us to evaluate participants’ performance across a continuum of cognitive demands, reflecting varying degrees of working memory involvement, and the study focuses on two distinct cognitive domains: the visuospatial and the linguistic. These domains were selected to examine the extent to which performance on the spoonerism task is influenced by domain-specific versus domain-general systems. The children were individually tested in a quiet room at their school. The battery of tests was administered on different days, each session lasting approximately one hour. Each child had different abilities and required a different amount of time to complete the tasks. This implied that the total duration of task sessions varied according to the individual child’s abilities. Below is a description of the different measures used.

### 2.3. Measures

#### Working Memory Capacity Tasks

Task 1: The alpha span test (AST, from the MEA Battery, [49]) assessed alphabetical knowledge and WMC. Children were presented with a list of words and asked to repeat them in the same order (passive span) and then in alphabetical order (active span). This task measured executive attention and the simultaneous storage and manipulation of information without relying on chunking or repeating strategies. It aligned with the revised model of Baddeley and the concept of working memory capacity [50] as a set of non-separable executive functions.

Task 2: The object updating task (OUT, [51]) revised by [45] involved children listening to lists of words consisting of abstract and concrete words. They were asked to repeat three words corresponding to specific categories (tools, animals, vegetables, etc.) and then to repeat the names of two smaller objects. This task assesses auditory attention, short-term memory, imaginative skills, and WMC. The task used blocks of three lists, increasing in length if the child responded correctly, and the number of words in the last list that were repeated correctly was recorded.

Task 3: In the spoonerism task (SPO, from the CMF Battery) [52], the children were asked to swap the initial phonemes of two words pronounced by an examiner or reproduced via loudspeakers to create, as quickly as possible, two new words (or non-words in some versions of the task). The task required segmenting the stimuli properly in order to identify the first phonemes (onsets) and the final part of the targets (rimes), maintaining this information temporarily, and then transposing the initial phonemes, blending the first onsets with the rime of the corresponding word (e.g., *Lack–Pies* becomes *Pack–Lies* after swapping the initial phonemes).

Task 4: In the verbal fluency task (VFT, from the CMF Battery, [52]), the children were asked to say aloud as many words as possible that began with a specific phoneme (/F/, /A/, and /S/). The time limit was one minute for each phoneme. The total number of correct words was measured. This task has been proposed to measure inhibitory functioning [36], memory monitoring [53], and switching between retrieval strategies [54].

Task 5: figural fluency task (FFT, from the MEA Battery, [49]) investigated the ability to generate ever-changing visual configurations in a unit of time, respecting certain rules provided before the task began. It consisted of four trials, each preceded by a training pre-test. The child was presented with configurations consisting of 5 dots; the task is (in each one) to join them in all possible ways in 1 min, respecting the following conditions: 2, 3, 4, or all 5 dots can be joined, but always different figures must be formed; no separate figures must be created; rows must always be straight and must touch the dots precisely.

Task 6: copying complex figures—the Rey’s figure (REY, [55]) was used to assess visual organization and visuoconstructive functions. In this task, participants were asked to copy a complex figure composed of several geometric elements.

Task 7: The developmental visual perception test (TPV, [56]) included a number of tasks aimed at assessing visual perception and visuomotor integration. In the present study, we used the “copy of figures” subtest. Children are asked to copy a number of geometric shapes that increase progressively in visual and motor complexity. For instance, early items might include simple shapes such as a square or triangle, while later items involve more intricate configurations with intersecting lines, asymmetry, or multiple embedded elements. As complexity increases, the task can begin to engage not only visual–motor coordination but also attentional control and executive resources (e.g., planning and monitoring the graphic gesture).

Task 8: forward enumeration task (FET, from the MEA Battery, [49]). Participants are asked to count aloud from 1 to 100, increasing by 1 with each number (i.e., 1, 2, 3, …). This task primarily involved automatic and sequential processing, with minimal reliance on WMC.

Task 9: rapid naming of numbers (RNN, [45]). Participants quickly named a series of visually presented numbers. This task measured automatic and rapid processing of numerical stimuli with minimal WMC demand. The automaticity of the “digit name” association, less evident in earlier years, naturally emerges in fourth graders who are now familiar with dealing with numbers.

## 3. Results

### 3.1. Step 1

To identify the high- and low-WMC groups, we employed two different tests that align with [50] WMC model: the verbal AST and a visuospatial object updating task (OUT). This approach used two different testing modes and was chosen to dispel any doubts regarding the specificity associated with updating tests, despite several studies confirming their general domain [50,57]. The verbal AST was taken from the MEA battery [49] for fourth-grade children and yielded a mean score of 3 and a standard deviation of 0.65. The low-WMC group consisted of 20 participants who scored between 0 and 2, with a score of 2 indicating approximately 1.5 standard deviations below the mean. The high-WMC group consisted of 29 participants who scored 4, which is approximately 1.5 standard deviations above the mean.

We also used the OUT that was specifically designed for this study and standardized it on the 115 participants (excluding 8 outliers). This test had a mean score of 3.90 and a standard deviation of 0.76. The low-WMC group consisted of 41 participants with scores ranging from 0 to 3, with a score of 3 representing approximately 1.18 standard deviations below the mean. The high-WMC group consisted of 23 participants with scores ranging from 5 to 6, which corresponded to 1.4 and 2.7 standard deviations above the mean, respectively.

The groups selected based on their results in the AST (20 low-WMC participants (span ≤ 2) and 29 high-WMC participants (span = 4)) were compared on measures of executive attention and WMC resources using independent sample *t*-tests, with Welch correction of the *t*-statistic and its associated degrees of freedom in the case of unequal variances. The *p*-values were adjusted for multiple comparisons using the Benjamini–Hochberg correction for false discovery rate (FDR). The possible biasing effect of outliers was ruled out by performing Mann–Whitney tests, which yielded the same results and are not reported here.

As shown in Table 2, participants in the low-WMC group struggled with more complex tasks, such as SPO, VFT, FFT, and REY, while no substantial differences were observed in tests that are more specific to the low-WMC load domain, such as FET and RNN. The effect size of the difference in the executive attention tests was moderate (0.50 ≤ *d* < 0.80) to high (*d* ≥ 0.80), indicating the need for WMC engagement. Both low- and high-WMC groups did not substantially differ (effect sizes low (0.20 ≤ *d* < 0.50) to moderate) in more automated linguistic tests, including modular tests, number naming, and rapid counting from 1 to 100, which do not require the activation of CEN circuits and thus WMC support.

The groups selected based on their results in the OUT (41 low-WMC participants (score ≤ 3) and 23 high-WMC participants (score ≥ 5)) were compared with the same strategy as above. The results are reported in Table 3 and showed large differences in SPO and FFT scores, a moderate, albeit marginally significant, difference after adjustment for FDR in VFT, and no substantial differences in the other modular linguistic tests.

### 3.2. Step 2

**Figure 2:**
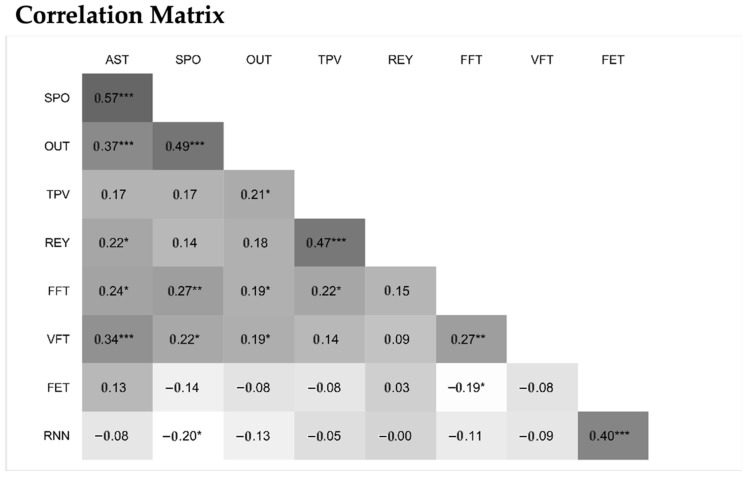
Correlation matrix for the measures used in this study (*n* = 115). ***: *p* < 0.001; **: *p* < 0.01; *: *p* < 0.05.

At this stage, we investigated the construct validity of the spoonerism task, i.e., whether it is more a linguistic test or a WMC test, by analyzing the correlation matrix of the scores of the tests administered in this study (Figure 2).

To rule out known methodological biases, we carefully examined the most appropriate model of analysis for investigating the construct of the spoonerism task relative to the testing context we used. We therefore considered the following:

A multiple regression model provides estimates of the contribution of each variable to the prediction of spoonerism while keeping the other variables constant. However, one or more variables can be predicted, in turn, by the other variables in the model, and this decreases the unique information that can be added to the prediction of the spoonerism task. This issue is known as “multi-collinearity” and tends to become more and more problematic when the multiple correlation of one or more variables with the set of other variables increases, making the estimates of single regression coefficients unreliable and difficult to interpret [58].

Although some strategies to address this issue have been proposed (see [58], Section 10.6), they cannot address another issue, namely, “predictive mediation”. Predictive mediation occurs when two variables are not directly connected but are indirectly connected (e.g., A–B–C, where A, B, and C represent hypothetical variables). This indicates that A and C can be correlated, but any predictive effect from A to C (or vice versa) is mediated by B [59].

Gaussian graphical models (GGMs) have been proposed as a solution for both multi-collinearity and predictive mediation [60]. In a GGM, the observed variables are nodes of a network connected by edges that have partial correlation coefficients between the variables after conditioning on all other variables in the dataset. As a result, edges correspond to a multiple regression coefficient, i.e., an estimate of the strength of the direct association of one variable with another after controlling for all other variables in the network that rule out spurious effects due to other variables in the network.

Furthermore, the non-paranormal transformation [61] was used to address the relative non-normality of the distributions of test scores. As part of our analysis, we employed bootstrapping (10,000 samples) to identify edges that could be considered reliably different from zero, that is, their 95% confidence interval (2.5th–97.5th percentile) did not contain zero.

**Figure 3:**
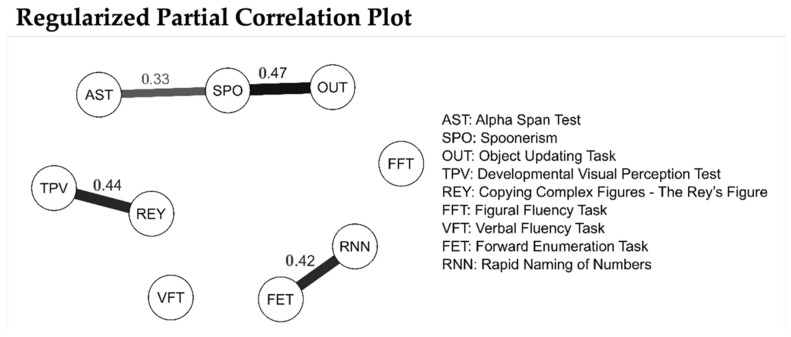
Regularized partial correlation plot for the measures used in this study (*n* = 115).

The results of the GGM are reported in Figure 3 and show that, once controlling for all other variables in the network, spoonerism is directly associated with object updating and alpha span, i.e., measures of WMC. Other non-zero direct associations were found between the visual perception test and complex figure copying and between enumeration and naming of numbers.

## 4. Discussion

The present study aimed to examine whether spoonerism tasks, commonly used to assess phonological awareness (PA), reflect a purely linguistic ability or a more complex cognitive construct involving domain-general systems such as working memory and executive attention. By analyzing performance in a sample of typically developing fourth graders, we sought to identify the cognitive components that most strongly predict success in a complex PA task.

Prior literature suggests that reading skills engage broader cognitive systems beyond phonological access alone [7,46,62,63]. Contrary to simpler phonological tasks (e.g., phoneme blending, segmentation that rely more heavily on phonological access and less on central executive systems), the spoonerism task appears to require the retrieval, temporary storage, manipulation, and recombination of phonemic information. This multi-step nature makes it particularly sensitive to the integrity of domain-general processes, such as executive attention and working memory, and multi-componential abilities, such as reading [64].

In general, our results of group comparisons showed that participants in the low-WMC group struggled more with complex tasks, while no difference was observed in tests requiring a lower WMC load. Specifically, when the groups were selected based on their performance in the alpha span test, significant group differences emerged in highly WMC-demanding tasks (namely spoonerism, verbal fluency, figural fluency, and Rey’s figure tasks), but not in more automated linguistic tests requiring low WMC support (like number naming and rapid counting). Similarly, when the groups were selected according to object updating performance, the results confirmed significant differences in spoonerism, verbal fluency, and figural fluency tasks, while no substantial differences emerged in the other linguistic tests.

In addition to these findings, the results of the GGM showed that performance in the spoonerism task was strongly associated with the object updating task (0.47) and the alpha span test (0.33), confirming the close affinity between this phonological awareness task and the components of working memory, rather than merely linguistic skills.

This finding is consistent with the framework proposed by Engle and colleagues [65,66,67], who conceptualize working memory capacity as reflecting domain-general attentional control that operates over domain-specific representations. For instance, digit span forward primarily measures immediate verbal memory span and sequencing [68,69], but as the span length increases, the involvement of executive control processes also increases [70]. The Alpha Span Test, which requires not only retention but also reordering of items, places demands on executive attention and the updating component of working memory [50,71,72]. The object updating task involves ordering verbal items based on size, drawing on executive control, auditory attention, lexical–semantic knowledge, and visuospatial working memory [73,74,75]. The predictive value of these three tasks suggests that the performance on the spoonerism task relies on the ability to coordinate linguistic and executive resources under increasing cognitive load.

To interpret these findings, we refer to recent theories of dynamic modularity proposed by Benso et al. (2025) [39], which build on and expand beyond the earlier proposals by Moscovitch and Umiltà (1990) [38]. This framework conceptualizes modularity not as an anatomically fixed feature but as a functional state dependent on task demands and automatization. These higher-level systems may achieve modular-like properties with extensive practice but continue to rely on executive attention, particularly in novel or demanding contexts [39].

The spoonerism task, although traditionally considered a phonological assessment, requires the integration of visual, phonological, and executive components. To perform the task, participants must mentally hold and manipulate orthographic representations, swap initial phonemes, and articulate the transformed sequence—operations that draw heavily on working memory and attentional control. Unlike reading, which may automatize with experience and develop modular features, spoonerism remains effortful and non-automatizable, even in expert readers.

This interpretation helps explain the strong associations observed between spoonerism performance and both verbal and visuospatial working memory in our study. Rather than measuring phonological awareness in isolation, spoonerism reflects a multi-componential construct—a convergence of domain-general and domain-specific processes mediated by central executive systems. From this perspective, it serves as a useful proxy for studying how cognitive systems integrate and evolve toward functional specialization.

These findings align with contemporary views of modularity as fluid and context-sensitive (e.g., [40,41,42]), rather than static or domain-encapsulated (cf. Fodorian models; [30,31,36,37,76]). For example, behaviors such as walking, which are typically automatic, can become effortful under uncertain conditions (on a narrow and unstable mountain path requiring continuous cognitive adjustments; [39,41]), reflecting a temporary loss of mandatoriness, that is, a temporary loss of modular status. Likewise, spoonerism elicits high executive demand, indicating that its cognitive architecture does not support a modular classification.

Finally, following Dehaene and Cohen (2011) [47], we prefer the term functional specialization over domain specificity for third-level systems, as this captures the integrative, overlapping nature of higher cognitive functions. Taken together, these observations support a re-evaluation of complex phonological assessments like spoonerism. While not modular, spoonerism mirrors the transitional architecture of systems moving toward modularity and, as such, provides valuable insight into the interaction between language and executive control in developing readers.

Taken together, our findings underscore the importance of re-evaluating complex phonological assessments like the spoonerism task through the lens of dynamic modularity, offering new insights into the interplay between linguistic and executive systems during reading development.

## 5. Limitations and Future Perspectives

The results of this study contribute to highlighting the complex nature of a multi-componential task, the spoonerism task, that has long been considered as a purely linguistic measure. However, as a limitation we acknowledge that in the present study we focused on a narrow age range (i.e., fourth graders) in typically developing children and we did not include a measure of reading proficiency. Therefore, further research is desirable to validate and generalize our results in a clinical population, e.g., learning difficulties, and different ages.

Another possible limitation may be the absence, within our neuropsychological battery, of a specific test tailored to assess executive attention per se. However, the primary objective of our study was not to obtain a direct measure of executive attention by means of a dedicated test but rather to include a variety of verbal and non-verbal tasks with progressively increasing executive demand. The choice of including different visuospatial and verbal tasks characterized by escalating cognitive load is consistent with the theoretical background underpinning the present study, namely the modularity framework proposed by Benso et al. (2025) [39] and grounded in the work of Moscovitch and Umiltà (1990) [38]. Within this framework, we aimed to investigate the multi-componential architecture of spoonerism including both domain-specific and domain-general components, encompassing verbal and non-verbal domains.

Although the lack of correlation with linguistic tasks may suggest a limited role for purely phonological processing, this cannot be definitively concluded. Since the null hypothesis cannot be rejected, the possibility remains open that different or more cognitively demanding linguistic tasks might yield significant associations—thus potentially falsifying the current findings. This reflects the methodological caution required when interpreting non-significant results.

Despite these limitations, our findings contribute to the limited body of research on the role of working memory capacity in complex tasks and offer a valuable starting point for future investigations of this relation. Moreover, our results have implications not only from a theoretical point of view but also from an educational and clinical point of view. Indeed, understanding the influence of broad cognitive components on linguistic skills and reading abilities can guide the development of more effective and personalized interventions for reading disorders, such as dyslexia.

## 6. Conclusions

This study set out to determine whether performance on the spoonerism task reflects a purely linguistic ability or a more complex, multi-componential construct involving domain-general systems such as working memory and executive attention. Our findings support the hypothesis that spoonerism task performance is best understood as the result of an interaction between linguistic executive attention and working memory processes, rather than as a measure of phonological awareness alone. This hypothesis is substantiated by two converging lines of evidence that reinforce one another: (1) the recently proposed modular theory [39], which frames the spoonerism task as a “non-module”; and (2) results of our statistical analyses indicating the multi-factorial nature of the task, with a particularly strong association with working memory functions.

These findings have both theoretical and applied implications. Theoretically, they prompt a broader reconsideration of metaphonological tasks, raising the question of whether other PA measures also engage executive systems. This aligns with recent evidence showing that both phonological and non-phonological factors contribute to early literacy acquisition [77].

While spoonerism tasks are widely used in predicting developmental dyslexia (e.g., [12,46,78,79]), their validity as purely linguistic indicators warrants scrutiny. The demands of these tasks—such as sequencing, inhibition, and memory updating—challenge the assumption that they assess phonological skills in isolation. If they are inherently multi-componential, interpreting poor performance as a phonological deficit alone may be misleading (see also [80], for a critique of the phonological deficit theory’s explanatory power and testability).

This raises broader questions in dyslexia research: is language the primary bottleneck, or do executive and attentional systems play a more central role than traditionally acknowledged? Revisiting long-established models (e.g., [17,81,82]) through a more integrative lens could lead to more accurate diagnostic and intervention frameworks.

From an applied perspective, spoonerism tasks may offer added diagnostic value beyond what simpler PA or WM tasks reveal. Their hybrid nature makes them particularly useful for identifying children whose reading difficulties stem from executive dysfunctions rather than—or in addition to—phonological deficits. This has practical relevance for educational and clinical settings, where personalized intervention strategies can benefit from a clearer understanding of a child’s full cognitive profile.

In sum, the spoonerism task offers a window into the dynamic interplay between language and cognition. Its continued use in research and practice is warranted—provided that we interpret its results in light of the multiple cognitive systems it engages.

## Figures and Tables

**Table 2:** Comparisons of High and Low Working Memory Capacity (WMC) groups’ performance on the Alpha Span Test.

	Group								Statistics			
Response Variable	Low-WMC				High-WMC							
	n	M	SD	Range	n	M	SD	Range	t(df)	*p*	adj-*p*	d
SPO	20	14.45	7.92	1-29	29	26.28	3.57	17–30	−6.257 (24.36)	<0.001	<0.001	1.92
VFT	20	16.95	6.09	9–29	29	25.31	7.25	9–42	−4.266 (47)	<0.001	<0.001	1.28
FFT	19	29.42	12.73	11–47	29	40.76	12.76	12–64	−3.012 (46)	0.004	0.010	0.73
REY	20	26.93	5.39	14.50–35	29	30.66	3.94	20.50–36	−2.801 (47)	0.007	0.013	0.79
TPV	20	33.60	3.52	26–38	29	35.76	3.64	25–40	−2.068 (47)	0.044	0.062	0.60
FET	20	63.64	9.74	49–88	29	68.02	10.38	48–94	−1.489 (47)	0.143	0.167	0.43
RNN	20	14.90	4.17	9–23	29	14.17	3.30	10–23	0.682 (47)	0.499	0.499	0.20

*Note*: *n* = sample size; *M* = mean; *SD* = standard deviation; t(df) = *t*-statistic and its degrees of freedom; *p* = *p*-value; *adj-p* = Benjamini–Hochberg *p*-value adjustment for false discovery rate; *d* = Cohen’s measure of effect size. SPO = Spoonerism Task; VFT = Verbal Fluency Task; FFT = Figural Fluency Task; REY = Copying Complex Figures—The Rey’s Figure; TPV = Developmental Visual Perception Test; FET = Forward Enumeration Task; RNN = Rapid Naming of Numbers.

**Table 3:** Comparisons of High and Low Working Memory Capacity (WMC) groups based on the performance on the Object Updating Task.

	Group								Statistics			
Response Variable	Low-WMC				High-WMC							
	n	M	SD	Range	n	M	SD	Range	t(df)	*p*	adj-*p*	d
SPO	41	17.61	6.98	1–29	23	27,87	2.47	20–30	−8.510 (54.93)	<0.001	<001	1.96
VFT	41	19.07	5.76	9–34	23	23.04	8.05	9–42	−2.287 (62)	0.026	0.060	0.57
FFT	40	32.45	12.64	11–51	23	42.30	9.55	23–59	−3.493 (56.37)	0.001	0.003	0.88
REY	41	28.11	5.12	14.50–36	23	28.78	4.57	17.5–36	−0.524 (62)	0.602	0.602	0.14
TPV	41	35.02	2.98	27–40	23	35.83	3.66	26–40	−0.950 (62)	0.346	0.484	0.24
FET	41	65.22	10.10	49–94	23	63.44	12.99	42–94	0.609 (62)	0.545	0.602	0.15
RNN	41	15.24	4.13	9–28	23	14.22	3.07	10–20	1.039 (62)	0.303	0.484	0.28

*Note*: *n* = sample size; *M* = mean; *SD* = standard deviation; t(df) = *t*-statistic and its degrees of freedom; *p* = *p*-value; *adj-p* = Benjamini–Hochberg *p*-value adjustment for false discovery rate; *d* = Cohen’s measure of effect size. SPO = Spoonerism Task; VFT = Verbal Fluency Task; FFT = Figural Fluency Task; REY = Copying Complex Figures—The Rey’s Figure; TPV = Developmental Visual Perception Test; FET = Forward Enumeration Task; RNN = Rapid Naming of Numbers.

## Data Availability

Data and R scripts used are available on OSF: https://osf.io/uvawz/?view_only=1a7d12293c094aadb85b4cb230ed4494.

## References

[1] Wagner R.K., Torgesen J.K. (1987). The Nature of Phonological Processing and Its Causal Role in the Acquisition of Reading Skills. Psychol. Bull..

[2] Anthony J.L., Francis D.J. (2005). Development of Phonological Awareness. Curr. Dir. Psychol. Sci..

[3] Ziegler J.C., Goswami U. (2005). Reading Acquisition, Developmental Dyslexia, and Skilled Reading Across Languages: A Psycholinguistic Grain Size Theory. Psychol. Bull..

[4] Anthony J.L., Lonigan C.J. (2004). The Nature of Phonological Awareness: Converging Evidence from Four Studies of Preschool and Early Grade School Children. J. Educ. Psychol..

[5] Mann V., Sawyer D.J., Fox B.J. (1991). Phonological Awareness and Early Reading Ability: One Perspective. Phonological Awareness in Reading.

[6] Ball E.W. (1993). Phonological awareness—What’s important and to whom?. Read. Writ..

[7] Knoop-van Campen C.A.N., Segers E., Verhoeven L. (2018). How phonological awareness mediates the relation between working memory and word reading efficiency in children with dyslexia. Dyslexia.

[8] Gray S., Green S., Alt M., Hogan T., Kuo T., Brinkley S., Cowan N. (2017). The structure of working memory in young children and its relation to intelligence. J. Mem. Lang..

[9] Baddeley A., Hitch G., Allen R. (2020). A Multicomponent Model of Working Memory. Working Memory: State of the Science.

[10] Hitch G.J., Allen R.J., Baddeley A.D. (2025). The multicomponent model of working memory fifty years on. Q. J. Exp. Psychol..

[11] Bradley L., Bryant P.E. (1983). Categorizing sounds and learning to read-A causal connection. Nature.

[12] Snowling M.J. (2009). Dyslexia. https://www.wiley.com/en-us/Dyslexia%2C+2nd+Edition-p-9780631205746.

[13] Vellutino F.R., Fletcher J.M., Snowling M.J., Scanlon D.M. (2004). Specific reading disability (dyslexia): What have we learned in the past four decades?. J. Child. Psychol. Psychiatry Allied Discip..

[14] Melby-Lervåg M., Lyster S.A.H., Hulme C. (2012). Phonological skills and their role in learning to read: A meta-analytic review. Psychol. Bull..

[15] Scarborough H.S. (1990). Very Early Language Deficits in Dyslexic Children. Child. Dev..

[16] Catts H.W., Fey M.E., Zhang X., Tomblin J.B. (2001). Estimating the Risk of Future Reading Difficulties in Kindergarten Children. Lang. Speech Hear. Serv. Sch..

[17] Ramus F. (2003). Developmental dyslexia: Specific phonological deficit or general sensorimotor dysfunction?. Curr. Opin. Neurobiol..

[18] Pennington B.F. (2006). From single to multiple deficit models of developmental disorders. Cognition.

[19] Valdois S., Bosse M.L., Tainturier M.J. (2004). The cognitive deficits responsible for developmental dyslexia: Review of evidence for a selective visual attentional disorder. Dyslexia.

[20] Bosse M.L., Tainturier M.J., Valdois S. (2007). Developmental dyslexia: The visual attention span deficit hypothesis. Cognition.

[21] Di Filippo G., Zoccolotti P., Ziegler J.C. (2008). Rapid naming deficits in dyslexia: A stumbling block for the perceptual anchor theory of dyslexia. Dev. Sci..

[22] Lallier M., Tainturier M.J., Dering B., Donnadieu S., Valdois S., Thierry G. (2010). Behavioral and ERP evidence for amodal sluggish attentional shifting in developmental dyslexia. Neuropsychologia.

[23] Grainger J., Lété B., Bertand D., Dufau S., Ziegler J.C. (2012). Evidence for multiple routes in learning to read. Cognition.

[24] Ziegler J.C., Castel C., Pech-Georgel C., George F., Alario F.X., Perry C. (2008). Developmental dyslexia and the dual route model of reading: Simulating individual differences and subtypes. Cognition.

[25] Pennington B.F., Santerre-Lemmon L., Rosenberg J., MacDonald B., Boada R., Friend A., Leopold D., Daniel R., Samuelsson S., Byrne B. (2012). Individual prediction of dyslexia by single versus multiple deficit models. J. Abnorm. Psychol..

[26] Peterson R.L., Pennington B.F. (2015). Developmental dyslexia. Annu. Rev. Clin. Psychol..

[27] Castles A., Coltheart M. (2004). Is there a causal link from phonological awareness to success in learning to read?. Cognition.

[28] Oakhill J., Kyle F. (2000). The Relation between Phonological Awareness and Working Memory. J. Exp. Child. Psychol..

[29] Fodor J.A. (1983). The Modularity of Mind.

[30] Anderson M.L. (2010). Neural reuse: A fundamental organizational principle of the brain. Behav. Brain Sci..

[31] Barrett H.C., Kurzban R. (2006). Modularity in cognition: Framing the debate. Psychol. Rev..

[32] Ritchie J.B., Carruthers P. (2010). Massive modularity is consistent with most forms of neural reuse. Behav. Brain Sci..

[33] Rizzolatti G., Craighero L. (2004). The mirror-neuron system. Annu. Rev. Neurosci..

[34] Wagner J.B., Hirsch S.B., Vogel-Farley V.K., Redcay E., Nelson C.A. (2013). Eye-tracking, autonomic, and electrophysiological correlates of emotional face processing in adolescents with autism spectrum disorder. J. Autism Dev. Disord..

[35] Chen L., Wassermann D., Abrams D.A., Kochalka J., Gallardo-Diez G., Menon V. (2019). The visual word form area (VWFA) is part of both language and attention circuitry. Nat. Commun..

[36] Mahon B.Z., Cantlon J.F. (2011). The specialization of function: Cognitive and neural perspectives. Cogn. Neuropsychol..

[37] Rabaglia C.D., Marcus G.F., Lane S.P. (2011). What can individual differences tell us about the specialization of function?. Cogn. Neuropsychol..

[38] Moscovitch M., Umilta C. (1990). Modularity and Neuropsychology: Modules and Central Processes in Attention and Memory. Modular Deficits in Alzheimer-Type Dementia.

[39] Benso F., Chiorri C., Ardu E., Venuti P., Pasqualotto A. (2025). Beyond modular and non-modular states: Theoretical considerations, exemplifications, and practical implications. Front. Psychol..

[40] Dosenbach N.U.F., Fair D.A., Miezin F.M., Cohen A.L., Wenger K.K., Dosenbach R.A., Fox M.D., Snyder A.Z., Vincent J.L., Raichle M.E. (2007). Distinct brain networks for adaptive and stable task control in humans. Proc. Natl. Acad. Sci. USA.

[41] Hikosaka O., Isoda M. (2010). Switching from automatic to controlled behavior: Cortico-basal ganglia mechanisms. Trends Cogn. Sci..

[42] Menon V. (2015). Salience Network. Brain Mapp. Encycl. Academic Press..

[43] McCarthy K.M., Skoruppa K. (2023). Language-specific phonological skills and the relationship with reading accuracy in Sylheti-English sequential bilinguals. Child. Dev..

[44] Vender M., Melloni C. (2021). Phonological Awareness across Child Populations: How Bilingualism and Dyslexia Interact. Languages.

[45] Benso F., Clavarezza V., Caria A., Chiorri C. (2013). Validazione di un modello multicomponenziale della lettura. Dislessia-G. Ital. Ric. Clin. E Appl..

[46] Varvara P., Varuzza C., Sorrentino A.C.P., Vicari S., Menghini D. (2014). Executive functions in developmental dyslexia. Front. Hum. Neurosci..

[47] Dehaene S., Cohen L. (2011). The unique role of the visual word form area in reading. Trends Cogn. Sci..

[48] Anderson P. (2002). Assessment and development of executive function (EF) during childhood. Child. Neuropsychol..

[49] Benso F., Santoro G.M., Ardu E. (2019). MEA, Measures of Executive Attention. https://www.hogrefe.it/i-nostri-progetti/lavori-corso/mea-measures-of-executive-attention/.

[50] Engle R.W., Kane M.J. (2004). Executive Attention, Working Memory Capacity, and A Two-Factor Theory of Cognitive Control. Psychol. Learn. Motiv. Adv. Res. Theory.

[51] Palladino P., Cornoldi C., De Beni R., Pazzaglia F. (2001). Working memory and updating processes in reading comprehension. Mem. Cogn..

[52] Marotta L., Ronchetti Claudia Trasciani M., Vicari S. Test CMF-Valutazione Delle Competenze Metafonologiche. https://www.erickson.it/it/test-cmf-valutazione-delle-competenze-metafonologiche.

[53] Rosen V.M., Engle R.W. (1997). The Role of Working Memory Capacity in Retrieval. J. Exp. Psychol. Gen..

[54] Troyer A.K., Moscovitch M., Winocur G. (1997). Clustering and switching as two components of verbal fluency: Evidence from younger and older healthy adults. Neuropsychology.

[55] Lis A., Di Nuovo S. (1982). Test della grande figura complessa di Rey. Traduzione italiana. Organ. Spec. Firenze Italy.

[56] Hammill D., Pearson N., Voress J. Test TPV: Test Di Percezione Visiva e Integrazione Visuo-Motoria. https://www.ibs.it/tpv-test-di-percezione-visiva-libro-vari/e/9788879461146?srsltid=AfmBOor7-HjKhdHVIR1Qoz0xx0DV5asvCgCp75y7BKw-lnmNu2mmd_B7.

[57] Draheim C., Tsukahara J.S., Martin J.D., Mashburn C.A., Engle R.W. (2021). A toolbox approach to improving the measurement of attention control. J. Exp. Psychol. Gen..

[58] Cohen J., Cohen P., West S.G., Aiken L.S. (2013). Applied Multiple Regression/Correlation Analysis for the Behavioral Sciences.

[59] Epskamp S., Fried E.I. (2018). A tutorial on regularized partial correlation networks. Psychol. Methods.

[60] Epskamp S., Waldorp L.J., Mõttus R., Borsboom D. (2018). The Gaussian Graphical Model in Cross-Sectional and Time-Series Data. Multivariate Behav. Res..

[61] Liu H., Lafferty J., Wasserman L., Wainwright M.J. (2009). The Nonparanormal: Semiparametric Estimation of High Dimensional Undirected Graphs. J. Mach. Learn. Res..

[62] Beneventi H., TØNnessen F.E., Ersland L., Hugdahl K. (2010). Executive working memory processes in dyslexia: Behavioral and fMRI evidence. Scand. J. Psychol..

[63] Franceschini S., Gori S., Ruffino M., Pedrolli K., Facoetti A. (2012). A causal link between visual spatial attention and reading acquisition. Curr. Biol..

[64] Catts H.W., Petscher Y. (2022). A Cumulative Risk and Resilience Model of Dyslexia. J. Learn. Disabil..

[65] Conway A.R.A., Kane M.J., Bunting M.F., Hambrick D.Z., Wilhelm O., Engle R.W. (2005). Working memory span tasks: A methodological review and user’s guide. Psychon. Bull. Rev..

[66] Engle R.W., Tuholski S.W., Laughlin J.E., Conway A.R.A. (1999). Working memory, short-term memory, and general fluid intelligence: A latent-variable approach. J. Exp. Psychol. Gen..

[67] Kane M.J., Conway A.R.A., Bleckley M.K., Engle R.W. (2001). A controlled-attention view of working-memory capacity. J. Exp. Psychol. Gen..

[68] Baddeley A. (1992). Working memory. Science.

[69] Hale J.B., Hoeppner J.A.B., Fiorello C.A. (2002). Analyzing Digit Span Components for Assessment of Attention Processes. J. Psychoeduc. Assess..

[70] Craig M., Dewar M. (2018). Rest-related consolidation protects the fine detail of new memories. Sci. Rep..

[71] D’Esposito M., Postle B.R., Rypma B. (2000). Prefrontal cortical contributions to working memory: Evidence from event-related fMRI studies. Exp. Brain Res..

[72] Oberauer K., Süß H.M., Schulze R., Wilhelm O., Wittmann W.W. (2000). Working memory capacity-Facets of a cognitive ability construct. Pers. Individ. Dif..

[73] Morris N., Jones D.M. (1990). Memory updating in working memory: The role of the central executive. Br. J. Psychol..

[74] Schmiedek F., Hildebrandt A., Lövdén M., Wilhelm O., Lindenberger U. (2009). Complex Span Versus Updating Tasks of Working Memory: The Gap Is Not That Deep. J. Exp. Psychol. Learn. Mem. Cogn..

[75] Magimairaj B.M., Montgomery J.W. (2013). Examining the Relative Contribution of Memory Updating, Attention Focus Switching, and Sustained Attention to Children’s Verbal Working Memory Span. Child. Dev. Res..

[76] Carruthers P. (2007). The Architecture of the Mind.

[77] Pasqualotto A., Venuti P. (2025). Predictors of Reading and Spelling Difficulties in Italian Children: Specific Language and General Cognitive Skills. Learn. Disabil. Res. Pract..

[78] Tilanus E.A.T., Segers E., Verhoeven L. (2013). Diagnostic profiles of children with developmental dyslexia in a transparent orthography. Res. Dev. Disabil..

[79] Ding N., Peng P., Tang J., Ding Y., Zhao J. (2025). An investigation of phonological predictors in Chinese developmental dyslexia using a machine learning approach. Reading and Writing.

[80] Stein J.F., Zoccolotti P. (2022). Success Is Not the Entire Story for a Scientific Theory: The Case of the Phonological Deficit Theory of Dyslexia. Brain Sci..

[81] Ramus F., Szenkovits G. (2008). What phonological deficit?. Q. J. Exp. Psychol..

[82] Lyytinen H., Guttorm T.K., Huttunen T., Hämäläinen J., Leppänen P.H.T., Vesterinen M. (2005). Psychophysiology of developmental dyslexia: A review of findings including studies of children at risk for dyslexia. J. NeuroLinguist..

